# Implementation of a Regional STEMI Network in North Cairo (Egypt): Impact on The Management and Outcome of STEMI Patients

**DOI:** 10.5334/gh.1182

**Published:** 2023-01-23

**Authors:** Sameh M. Shaheen, Atef K. Saleh, Nireen K. Okasha, Mohammed A. Abdalhamid, Hany M. Fakhry, Ramez R. Guindy

**Affiliations:** 1 Cardiology Department, Faculty of Medicine, Ain Shams University, EG; 2 Cardiology Department, Nasser Institute, Cairo, EG

**Keywords:** ST elevation Myocardial Infarction (STEMI), STEMI network, Egypt

## Abstract

**Background:**

Regional ST-segment–elevation myocardial infarction (STEMI) networks facilitate timely performance of primary percutaneous coronary intervention (PPCI), reduce mortality and improve outcomes. Few data exist on the feasibility and impact of regional STEMI networks in developing countries.

**Aim of the Work:**

The aim of this study was to examine the feasibility and impact of establishing a regional STEMI network on the management and outcomes of STEMI patients in north Cairo.

**Patients and Methods:**

A prospective observational study conducted on 352 patients presenting in North Cairo with confirmed diagnosis of STEMI within 48 hours of symptoms. Patients were divided into group I (n = 140) before and group II (n = 212) after establishment of the STEMI network. Both groups were compared as regards patients’ demographics, presentation, management and short-term outcomes. The north Cairo regional STEMI network was established among four governmental hospitals and the governmental ambulance was used for interhospital transfer. WhatsApp® was used for trans-network team communication.

**Results:**

Mean age of the study population was 55.4 ± 11.02 years and 286 (81.3%) were males. Mean time from chest pain to first medical contact did not change between the two groups (240 minutes; P = 0.36) while door to balloon mean time was reduced (from 54.3 to 44.1 minutes: P = 0.01). Use of thrombolytic therapy declined from 51 (36.4%) to 16 (7.5%) (P < 0.001) while primary PCI increased from 59.8% to 77.1% (p < 0.001). Left ventricular ejection fraction improved from 51.3 ± 10.7 to 55.4 ± 9.1 (P < 0.001), the mean time of CCU stay was reduced from a mean of 3.0 to 2.0 days (P < 0.001) and in-hospital mortality improved from 6.4% to 2.8% (P = 0.10).

**Conclusion:**

The establishment of the STEMI network in north Cairo was feasible and improved patients’ outcomes. Use of primary PCI increased and in-hospital mortality improved from after establishment of STEMI network.

## Introduction

Acute ST-segment elevation myocardial infarction (STEMI) is the most serious manifestation of coronary artery disease (CAD) with high morbidity and mortality [1]. Mortality due to CVD in Egypt is one of the highest compared to other countries in the region and worldwide [2]. Optimal treatment of STEMI patients in developed countries is based on the establishment of regional networks among primary PCI-capable and non-capable hospitals connected by an efficient ambulance service. The goal of these networks is to provide optimal care while minimizing delays, in order to improve clinical outcomes. The network depends on a hub and spoke system, where the hub performs primary PCI to eligible STEMI patients on a 24/7 basis, whereas the non-PCI-capable hospitals (or the spokes) transfer STEMI patients to the hub either with or without prior thrombolytic therapy [3]. Outcome of STEMI patients depends on the availability of emergency medical services provided by STEMI networks and the time delay before which a patient is treated [4]. Some studies have shown that obtaining an electrocardiogram (ECG) before reaching the hospital significantly reduces the door-to-balloon time, and that subsequently admitting ambulances directly to the cardiac catheterization laboratory (CCL), bypassing the emergency room (ER), substantially reduces the time to primary PCI resulting in a significant time saving for patients [5]. Prehospital activation has led to preserved ejection fractions, the shortest hospital stays [6] and reduction in mortality too [7]. Few data exist on the feasibility and impact of regional STEMI networks in developing countries. There are no well-organized regional STEMI networks in Egypt, despite having many large tertiary hospitals that are PPCI-capable around the clock. The establishment of such networks, if proved feasible and effective, could be a model to other similar networks all over the country and in other developing countries.

## Aim of the Work

The aim of this study was to assess the feasibility of establishing a regional STEMI network in north Cairo and to assess its impact on the management of STEMI patients and on their outcome.

## Patients and Methods

### Patient inclusion criteria

Patients diagnosed as STEMI should have the following criteria: chest pain lasting for at least 20 minutes and not relieved by rest or nitrates, symptom onset within the last 48 hours, have ST-segment elevation of at least 0.1 mV in at least two limb leads, ST-segment elevation of at least 0.1 mV in two or more contiguous precordial leads; or recent left bundle branch block (LBBB) or right bundle branch block (RBBB), relevant increase in myocardial biomarkers, eligible for reperfusion either by primary percutaneous coronary intervention (PPCI) or by thrombolytic therapy [8], and admitted to the north Cairo governorate hospitals during the period from the 1^st^ of October 2018 until the 30^th^ of September 2019.

### Study design

This was a prospective multicenter cross-sectional observational study. We developed a network based on a hub and spoke model with one main PPCI-capable hospital acting as a central hub and three smaller nearby hospitals acting as referring spokes to the hub. These four centers serve a population of 561,816 in north Cairo [9]. Using an accessible digital communication platform (WhatsApp®), a tele-communication group was initiated and included all doctors dealing with STEMI patients in these four hospitals together with the head of EMS in north Cairo. This group allowed sending patient data and remote analysis of ECGs of patients with suspected STEMI. Once a STEMI patient arrives at the spoke, an immediate ECG was transmitted to the hub. Once the diagnosis is confirmed, and if a bed is available, the patient was transferred urgently by the EMS from the spoke to the hub to receive PPCI. In the hub, the patient was transferred directly to the CCL bypassing the ER and coronary care unit (CCU). In case of an expected delay, the patient was given thrombolytic therapy then transferred to the hub within 2–24 hours. A written protocol was applied by the hub, spokes and EMS. An EMS system was activated once a STEMI patient was diagnosed and a decision to transfer to the hub was taken. Data were collected in regards to patients’ characteristics, mode of presentation, timing and mode of reperfusion, hospital stay and in-hospital outcomes. Patients were divided into two groups; Group I: those admitted in the first six months before establishing the network; and Group II: those admitted in the second six months after establishing the network. The two groups were compared to test the impact of such network on management and outcomes.

The primary PCI-capable hospital (the hub); Nasser Institute: 850 inpatient beds, 109 ICU beds including eight CCU beds, with three CCL facilities, two of them for coronary intervention and one for electrophysiological studies and pacemakers. Cardiac catheterization laboratory Volume: >3500 angiographic and interventional procedures/year, the primary PCI operators are qualified with availability of cardiology resident and specialist in ER over 24 hours. The non-PCI-capable hospitals (the spokes); Shoubra General Hospital: 182 inpatient beds, 28 ICU beds with cardiology specialist at the working hours and cardiology or critical care resident over 24 hours. Road El Farag General Hospital: 22 inpatient beds and 6 CCU beds with internist or critical care resident 24 hour service. El-Khazindara General Hospital: 67 working beds and 11 ICU beds with internist or critical care resident 24 hour service. A questionnaire was applied on the resources available in each hospital for the care of these patients. Two training sessions were organized to doctors, nurses and technicians in the four hospitals to demonstrate the value of early diagnosis and rapid referral of patients with STEMI to a hub hospital. Training was carried out on how to perform quality ECG, which allows the correct diagnosis. A flow chart was used containing the guidelines on sending the ECG via WhatsApp® when a STEMI was suspected and the transfer of these patients to the hub hospital.

### Data management and statistics

Data were collected then revised, coded, and entered to the statistical package for social science (SPSS) version 17. Qualitative data were presented as number and percentages while quantitative data were presented as mean, standard deviations and ranges. The comparison between two groups with qualitative data was done by using Chi-square test and Fisher exact test was used only when the expected count in any cell was found to be less than five. The comparison between two groups with quantitative data was done by using independent t-test when the data were parametric and Mann-Whitney test when the data were non parametric. This study was approved by the Research Ethics Committee of the four hospitals and followed the recommendations of the Helsinki declaration.

## Results

### Study population

The study was conducted on 352 patients who presented to four hospitals in north Cairo. The mean age of the study population was 55.40 ± 11.02 years and the majority of the patients were males (81.3%). Smoking was the predominant risk factor (60.2%) and 13.4% of the patients were drug addicts. The majority of patients (56.8%) presented by anterior myocardial infarction and 4.8% presented by symptoms of Killip Class IV. The mean left ventricular Ejection fraction was 53.8 ± 10.0, significant mitral regurgitation was diagnosed in three patients (0.9%) and ventricular septal rupture in one patient (0.3%). In this study, 274 patients (77.8%) had PCI with drug-eluting stents while 67 patients (19%) received streptokinase; of whom 12 had a pharmaco-invasive approach, six had rescue PCI and 49 patients had stand-alone thrombolysis. Aspiration thrombectomy was used in 6 patients (2.2%) only while IIb IIIa inhibitors were used in 67 patients (22.1%). The 2PY12 inhibitor used was clopidogrel in 90.1% of the patients while it was ticagrelor in 9.9%. The femoral access was predominant (98.1%) and LAD was the most common infarct related artery (54.1%). CABG was offered to 11 patients and in four patients the medical treatment was the preferred option. MINOCA was diagnosed in six patients. Only 3.1% of the patients were referred back to the spokes after intervention. Coronary dissection occurred in one patient (0.3%), perforation in two (0.6%), major bleeding in seven (2.0%), reinfarction in one patient (0.3%), stroke in two (0.6%), and stent thrombosis in two (0.6%). In-hospital mortality was declared in 15 patients (4.3%).

### Comparison between the study groups

**Figure 1:**
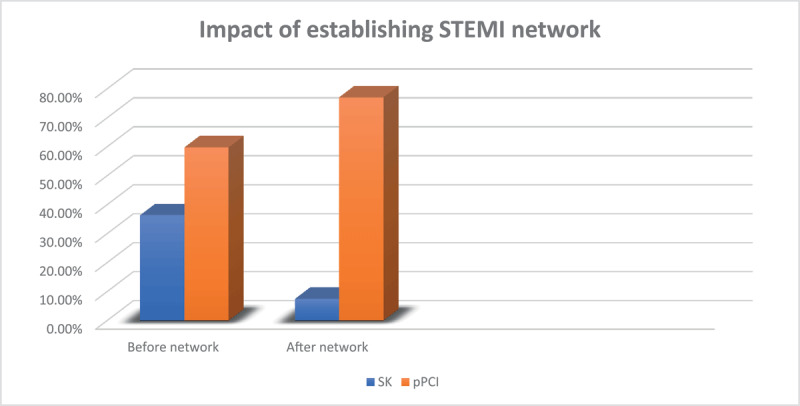
Impact of establishing STEMI network on the study patients’ management.

**Figure 2:**
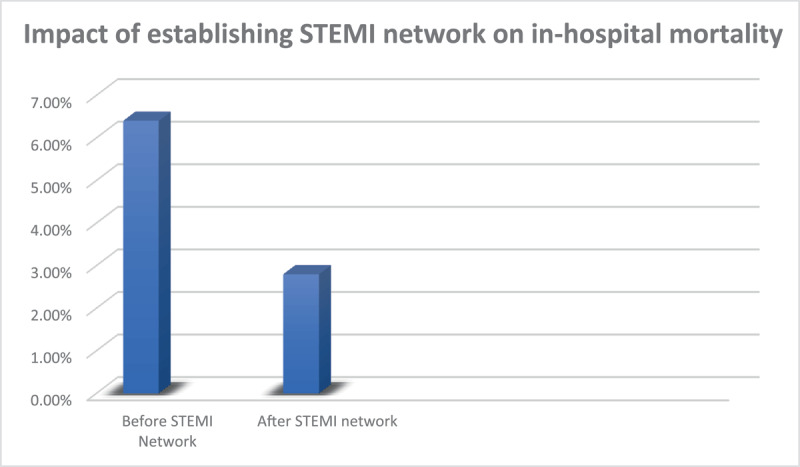
Impact of establishing STEMI network on in-hospital mortality.

The 352 patients were divided into two groups: Group I (140 patients) in the six months before and Group II (212 patients) in the six months after the network was established (Table 1). There was no statistically significant difference between both groups in regards to age and gender (P = 0.34 and 0.83; respectively). There was no statistically significant difference between both groups as regards risk factors except for obesity and peripheral arterial disease (PAD) which were more common in group I (P = 0.002 and 0.08; respectively). The median time from chest pain onset to first medical contact (FMC) was four hours in both groups. Door-to- balloon time was 54.3 min in group I and improved to 44.1 min in group II (p = 0.01) while door-to-needle time in both groups were not significantly different (33.2 min vs. 32.2 min; p = 0.81). The median total ischemic time also was unchanged in both groups (P = 0.48). Use of streptokinase dropped significantly after implementation of STEMI network from 36.4% to 7.5% (p < 0.001) while Primary PCI increased from 59.8% of the group I to 77.1% of group II (P =< 0.002) (Figure 1). The mean ejection fraction was 51.3% in the pre-implementation phase versus 55.4% in post-implementation (p < 0.001). Mitral regurgitation was more common in group I than group II (p < 0.001). Glycoprotein IIb IIIa inhibitors (Tirofiban) was given to 20.6% in group I versus 22.9% in group II (P = 0.649). The median CCU stay was three days before STEMI network establishment versus two days in the post implementation phase with significant difference (P < 0.001) and total hospital stay improved from five days in group I to four days in group II (P < 0.001). The median door-in-door-out time (DIDO) was 40 minutes after establishing the STEMI network. In-hospital mortality improved from 6.4% in group I to 2.8% in group II (P = 0.10) (Figure 2).

**Table 1:** Impact of establishing STEMI network on the study patients management and outcome.


		GROUP I (N = 140)		GROUP II (N = 212)		P VALUE

Chest pain to FMC (hr)	Median (IQR)	4.00	2.00–5.00	4.00	2.00–6.00	0.36

Total ischemic time (min)	Median (IQR)	270	171–420	270	200–420	0.48

Door to needle (min)	Mean (SD)	33.24	15.23	32.19	15.05	0.81

Door to balloon (min)	Mean (SD)	54.37	32.69	44.12	27.70	0.01

CCU stay (days)	Mean (SD)	3.0	2.0–4.0	2.0	2.0-3.0	<0.001

hospital stay (days)	Mean (SD)	5	4-6	4	3–4	<0.001

LVEF	Mean (SD)	51.3%	10.7	55.4	9.1	<0.001

Streptokinase	Number (%)	51	36.4%	16	7.5%	<0.001

Primary PCI	Number (%)	84	59.8%	163	77.1%	P =< 0.002

In-hospital mortality	Number (%)	9	6.4%	6	2.8%	0.10


## Discussion

Few data exist on the feasibility and impact of regional STEMI networks in developing countries. The current study was conducted to assess the feasibility and impact of establishing a STEMI network in north Cairo. The main outcome of the study was that the establishment of the STEMI network was feasible, improved the rate of performance of primary PCI, and reduced the rate of in-hospital mortality.

### Patients’ demographics

Egyptian STEMI patients were younger than their counterparts in developed countries. In this study, the mean age was 55.40 ± 11.02 years and 16.5% of the patients were ≤45 years. On the other hand, Waziri, et al. [10], found that myocardial infarction among patients ≤45 years was accounting for only 2–10% of all cases from the Eastern Danish Heart Registry and the Swedish Coronary Angiography and Angioplasty Registry. In our study, 60.2% of patients were smokers, 13.4% were drug addicts, 40.1% were diabetics, 41.0% were hypertensives, 10.5% were dyslipidemic and 11.4% were obese. The high prevalence of risk factors among Egyptian patients accounts for suffering from cardiovascular events at younger age and highlights the importance of developing a national prevention program.

### Prehospital management

In this study, patients delay before seeking medical advice did not improve by the establishment of the STEMI network; the median time from symptom onset to first medical contact (FMC) was 240 minutes before and after STEMI network establishment (P = 0.36). This was similar to an earlier Egyptian registry, in which the median time from symptom onset to first medical contact was significantly longer when compared to patients in European countries [2]. In this study, none of our STEMI patients presented to the hospitals via ambulance. This could be due to the close proximity of hospitals to the patients or the delay of EMS response in an overcrowded city. Similar results were reported in an earlier registry where EMS service was under-used (14%) among Egyptian patients presenting with STEMI [11]. Self-presentation rather than EMS-presentation and presenting to a non-PCI capable hospital are additional causes for pre-hospital delay in Egypt [2]. Such behavior needs public awareness campaigns to educate patients about possible heart attack symptoms and the recommended use of the EMS as the fastest and safest way to reach the dedicated hospital. Salerno, et al. [12] found no association between EMS call delay and education level, which supports the idea of mass awareness campaign regardless of the social level. In the French registries of Acute ST-elevation and non-ST-elevation myocardial infarction (FAST-MI), the reduction of the median time from symptom onset to admission from 240 min (in 1995) to 175 min (in 2010), resulted in a decrease in 30-day mortality [13].

### Interhospital transfer phase

In this study, the EMS service was the only method used for interhospital transfers from the spokes to the hub and the mean door-in-door-out time (DIDO) was 40 minutes. A benchmark of less than 30 minutes is recommended by the 2008 American College of Cardiology/American Heart Association (ACC/AHA) [14].

### Time of reperfusion

In our study, the mean door-to-balloon time was 54.3 ± 12.1 min before and improved to 44.1 ± 19.3 min after network establishment (P = 0.01). However, this was not much better than that reported by an earlier study by Yehia, et al. [15] in which 137 Egyptian STEMI patients were treated with primary PCI in a single tertiary center and their door-to-balloon time was 41 min. The mean door-to-needle time was 33.2 ± 15.5 min before and 32.2 ± 17.6 min after network set up (P = 0.81). In the Global Registry of Acute Coronary Events (GRACE), the median time to fibrinolysis was reduced from 40 min (in 1999) to 34 min (in 2006) (p < 0.001) but the door-to-balloon time remained unchanged (89 min vs. 87 min, P = 0.100) [16]. In a recent study, the establishment of a STEMI network helped to reduce the door-to-needle time. Egyptian health system needs more improvement to minimize pre-hospital and in-hospital delay in STEMI management [17].

### Mode of reperfusion

The rate of adopting primary PCI as the main mode of reperfusion is improving in Egypt. In the 2012 Egyptian STEMI Registry, 65.5% received streptokinase while only 12.4% had primary PCI [18]. In a more recent registry, the rate of Primary PCI was 49.12% while the rate of thrombolysis was 43.07% [11]. In the present study, the use of streptokinase declined from 36.4% to 7.5% (P > 0.001) after the implementation of the STEMI network. In our study, 285 PPCI was performed over one year in a region with 561,816 inhabitants; which means 507 PPCI per one million, a number approaching the recommendation of at least 600 PPCI per one million population. Routine coronary angiography with possible PCI to the infarct related artery within 2–24 h after thrombolytic therapy is often neglected in Egypt and streptokinase is still the only type of thrombolytics used.

A recent study from Slovakia aimed to assess the benefit of the systematic implementation of the new smartphone-based communication technology, STEMI, enabling immediate ECG picture and voice consultation between an EMS crew in the field and a cardiologist in the PCI center. The transfer of ECG was associated with 92% technical success. There was a significant decrease in unwanted secondary STEMI transportations (34.32% vs. 12.9%, p < 0.001) and a significant reduction in the total ischemic interval (241 min vs. 181 min, p = 0.03) [19].

### Hospital Stay

The establishment of a STEMI network helped to reduce the median CCU stay from 3.0 to 2.0 days (P > 0.001), and the total hospital stay from 5.0 to 4.0 days (P > 0.001). This helped to improve the availability of CCU beds for new patients.

### Mortality

In the present study, the overall in-hospital mortality was 4.3%. In an earlier study, in-hospital mortality rate among 137 patients treated with primary PCI in one Egyptian tertiary center was 6.5% [2]. In the current study, in-hospital mortality was reduced upon establishment of the STEMI network from 6.4% to 2.8%. As reported by Kristensen, et al. [20], the establishment of the STEMI Network was associated with a decreased in-hospital mortality. The same result was reported by García-García, et al. [21], when the STEMI network significantly increased the reperfusion rate (89.2% vs 64.4%, *p* < 0.001) and significantly reduced the in-hospital mortality (2.51% vs. 7.16%, *p* < 0.001).

In Brazil, 520 patients presenting with STEMI at 23 nonspecialized public health units or hospitals were enrolled in a prospective registry before and after the establishment of a regional STEMI network. Rates of primary reperfusion increased (29.1% vs.53.8%; P < 0.001) and more patients were transferred to the referral center (44.7% vs.76.3%; P = 0.001). Thirty-day mortality rates decreased from 19.8% to 5.1% (P < 0.001) [22].

Another study from Brazil analyzed outcomes before (82 patients) and after (196 patients) the organization of a telemedicine network to send the electrocardiogram via WhatsApp® of patients suspected of STEMI from 25 municipalities to a tertiary hospital. After implementing this network, there was a significant increase in the proportion of patients who received reperfusion therapy (60% vs. 92%; p < 0.0001) and decrease in the in-hospital mortality rate (13.4% vs. 5.6%; p = 0.028). According to the investigators of this study, the use of an accessible digital communication platform, such as WhatsApp®, with low-cost installation/maintenance that does not require training for its use, greatly facilitates its application and dissemination to other regions of the country [23].

A multicenter, prospective, observational study from South India, enrolled a total of 2,420 patients (2,034 men [84.0%] and 386 women [16.0%]; mean [SD] age, 54.7 [12.2] years) with symptoms or signs consistent with STEMI at primary care clinics, small hospitals, and PCI hospitals. Data were collected from four clusters before implementation of the program (pre-implementation data). The program was then implemented and data were then collected (post-implementation data) for a mean 32-week period. Patients (n = 898 in the pre-implementation phase and n = 1522 in the post-implementation phase) were enrolled, with 1,053 patients (43.5%) from the spoke health care centers. Overall reperfusion uses and times to reperfusion were similar (795 [88.5%] vs 1372 [90.1%]; *P* = 0.21). Coronary angiography (314 [35.0%] vs 925 [60.8%]; *P* < 0.001) and PCI (265 [29.5%] vs 707 [46.5%]; *P* < 0.001) were more commonly performed during the post-implementation phase. In-hospital mortality was not different (52 [5.8%] vs 85 [5.6%]; *P* = 0.83), but one-year mortality was lower in the post-implementation phase (134 [17.6%] vs 179 [14.2%]; *P* = 0.04). The authors concluded that a hub-and-spoke model in South India improved STEMI care through greater use of PCI and may improve one-year mortality. This model may serve as an example for developing STEMI systems of care in other low- to middle-income countries [3].

Investigators from Indonesia, compared the outcomes of STEMI patients before instalment of a STEMI network (N = 624), and five years after the start of the network (N = 1,052). Post-implementation, more primary PCI procedures were performed (N = 589 [56%] vs. N = 176 [28%], p < 0.001), fewer patients did not receive reperfusion therapy (37% vs. 59%, p < 0.001), and median door-to-device (DTD) times were shorter (82 vs. 94 minutes, p < 0.001). Overall, in-hospital mortality decreased from 9.6% to 7.1% (adjusted odds ratio 0.72, 95% CI: 0.50 to 1.03, p = 0.07)[24].

## Conclusion

The establishment of the STEMI network in north Cairo was feasible and improved patients’ outcomes. Use of primary PCI increased and in-hospital mortality improved from after establishment of STEMI network.
